# Letter from Rome, Italy

**Published:** 1890-03

**Authors:** 


### CORRESPONDENCE.

ROME, ITALY, Dec. 7th, 1889.

EDITOR OF REGISTER,

*Dear Sir*:—My hopes and promises to you to give something for the readers of the REGISTER during my journeyings abroad, jottings of what I saw and heard, have not been as fully realized as I fear you expected. But really circumstances beyond my control have so much diverted me from my original intention that many things that I proposed in the outstart have remained undone, and in attempting to now write I hardly know what to say.

Many things I saw and learned in England I could wish were otherwise. It really seems to me that the average European hardly realizes what we mean by dentistry. The amount of advertising by dentists in England is simply incredible, and altogether too often it is by Americans, but they are not the only sinners in this matter. I went into a barber shop in my wanderings about London, and was confronted with this singular advertisement, "Teeth cleaned and scaled by the head-barber."

On another occasion, when at Marseilles, in France, one evening I was watching the man on the opera house changing his glasses in his magic lantern, to project various views and advertisements on a large screen, on a building opposite. After a short time there was thrown on the screen the advertisement of a dentist. The first view was that of a man with his jaw bandaged and seemingly in great distress; the second, was the man in the dentist's chair; the third, was the dentist triumphantly showing an immense tooth supposed to have just been extracted from the man's jaw; fourth, price-list and address of the dentist. [Rubber plates six francs—$1.50].

These are only two of many such instances of charlatanism that fell in my way. I certainly did not seek them, for every such thing is an offense to every truly professional man. But all departments of human occupation have their shams and frauds, and our profession can hardly be expected to be a clean exception.

I will hope ere my travail is over to have something more acceptable to you and your readers. In the mean time, I am,

Yours truly, H. H. S.

